# Vaccinia Virus Protein Complex F12/E2 Interacts with Kinesin Light Chain Isoform 2 to Engage the Kinesin-1 Motor Complex

**DOI:** 10.1371/journal.ppat.1004723

**Published:** 2015-03-11

**Authors:** David C. J. Carpentier, William N. D. Gao, Helen Ewles, Gareth W. Morgan, Geoffrey L. Smith

**Affiliations:** 1 Department of Pathology, University of Cambridge, Cambridge, United Kingdom; 2 Department of Virology, Imperial College London, St. Mary’s Campus, London, United Kingdom; University of Wisconsin-Madison, UNITED STATES

## Abstract

During vaccinia virus morphogenesis, intracellular mature virus (IMV) particles are wrapped by a double lipid bilayer to form triple enveloped virions called intracellular enveloped virus (IEV). IEV are then transported to the cell surface where the outer IEV membrane fuses with the cell membrane to expose a double enveloped virion outside the cell. The F12, E2 and A36 proteins are involved in transport of IEVs to the cell surface. Deletion of the *F12L* or *E2L* genes causes a severe inhibition of IEV transport and a tiny plaque size. Deletion of the *A36R* gene leads to a smaller reduction in plaque size and less severe inhibition of IEV egress. The A36 protein is present in the outer membrane of IEVs, and over-expressed fragments of this protein interact with kinesin light chain (KLC). However, no interaction of F12 or E2 with the kinesin complex has been reported hitherto. Here the F12/E2 complex is shown to associate with kinesin-1 through an interaction of E2 with the C-terminal tail of KLC isoform 2, which varies considerably between different KLC isoforms. siRNA-mediated knockdown of KLC isoform 1 increased IEV transport to the cell surface and virus plaque size, suggesting interaction with KLC isoform 1 is somehow inhibitory of IEV transport. In contrast, knockdown of KLC isoform 2 did not affect IEV egress or plaque formation, indicating redundancy in virion egress pathways. Lastly, the enhancement of plaque size resulting from loss of KLC isoform 1 was abrogated by removal of KLC isoforms 1 and 2 simultaneously. These observations suggest redundancy in the mechanisms used for IEV egress, with involvement of KLC isoforms 1 and 2, and provide evidence of interaction of F12/E2 complex with the kinesin-1 complex.

## Author Summary

Viruses often hijack the cellular transport systems to facilitate their movement within and between cells. Vaccinia virus (VACV), the smallpox vaccine, is very adept at this and exploits cellular transport machinery at several stages during its life cycle. For instance, during transport of new virus particles to the cell surface VACV interacts with a protein motor complex called kinesin-1 that moves cargo on microtubules. However, details of the cellular and viral components needed and the molecular mechanisms involved remain poorly understood. Hitherto, only the VACV protein A36 has been shown to interact with kinesin-1, however viruses lacking A36 still reach the cell surface, albeit at reduced efficiency, indicating other factors are involved. Here we describe an interaction between kinesin-1 and a complex of VACV proteins F12 and E2, which are both needed for virus transport. The F12/E2 complex associates with a subset of kinesin-1 molecules (kinesin light chain isoform 2) with a region thought to be involved in modulation of cargo binding and kinesin-1 motor activity. Further study of this interaction will enhance understanding of the VACV life cycle and of the roles of different kinesin-1 subtypes in cellular processes and the mechanisms that regulate them.

## Introduction

Vaccinia virus (VACV) is a member of the *Orthopoxvirus* genus of the *Poxviridae* [1] and is the live vaccine that was used to eradicate smallpox [2]. Cells infected by VACV produce multiple structurally distinct forms of infectious virion [reviewed in 3,4]. The first assembles and matures within cytoplasmic viral factories [5], to form DNA-containing protein cores surrounded by a single lipid membrane [6,7] called intracellular mature virus (IMV) or mature virus (MV). Some IMVs are then wrapped by membranes derived from the trans-Golgi network or early-endosomes [reviewed in 4] to form the triple enveloped virion called intracellular enveloped virus (IEV) or wrapped virus (WV). IEV particles move to the cell periphery where the outer membrane fuses with the plasma membrane to expose a virion with 2 membranes outside the cell. Some of these virions are retained on the cell surface and are called cell-associated enveloped virus (CEV), and some are released into the extracellular matrix, called extracellular enveloped virus (EEV). The CEV and EEV forms have collectively also been called enveloped virus (EV) by some authors. Once on the cell surface, CEV particles induce the formation of actin tails to drive virions away from infected cells [8–13]. EEV particles mediate long range spread of virus. Actin tail formation is also exploited to enhance spread of VACV via the repulsion of superinfecting virions from infected cells [14,15].

Virus entry is mediated by a complex fusion machinery containing more than 10 virus proteins that promotes fusion of the virus envelope with the cell membrane (either at the cell surface [16,17], or after acidification within the endosome [18]) and results in the release of a naked virus core into the cytoplasm [reviewed in 19]. Cores migrate to a perinuclear location in a microtubule-dependent process [20], where they establish viral factories. IMVs formed in these factories are then transported on microtubules [21,22] to the site of wrapping and transport of IEVs to the cell periphery is also mediated by microtubules [8,23–26]. Naked virus cores, IMVs and IEVs each have different surfaces but all interact with the cellular microtubule trafficking machinery [reviewed in 3,4]. The mechanisms utilised by each of these particles and the cellular and virus proteins involved remain relatively incompletely understood. The most extensively studied trafficking event is the kinesin-1-mediated movement of IEVs from the site of wrapping to the cell surface [24].

Kinesin-1, or conventional kinesin, is a member of the kinesin superfamily of microtubule-associated motor proteins [reviewed in 27] that is involved in the intracellular trafficking of proteins, ribonucleoproteins and membrane bound organelles along microtubules away from the microtubule organising centre (MTOC). The kinesin-1 complex consists of a dimer of kinesin heavy chains (KHC), often, but not always [28], associated with two copies of a kinesin light chain (KLC). The KHC is composed of an amino-terminal microtubule-binding ATPase motor domain and a coiled-coil dimerisation domain. Three isoforms of KHC exist in mammals, Kif5A and Kif5C expressed in neurones, and Kif5B, which is expressed ubiquitously [29]. Some cargoes interact directly with KHC, such as the mitochondrial-associated protein milton [30], while others require the presence of KLCs. Four isoforms of KLC have been described in both humans and mice; the ubiquitous KLC2, the widely expressed but neuronal tissue enriched KLC1 [31], the testis-specific KLC3 [32] and KLC4, an isoform identified by bioinformatic screens of mouse and human genome sequences.

The envelopes acquired by VACV particles at the site of IMV wrapping to form IEV are associated with several VACV proteins [reviewed in 4]. These include proteins A33, A34, A36, A56, B5 and F13, of which B5 and F13 are required for IEV formation [33–35]. Two other proteins, F12 and E2, become associated with IEVs and are needed for transport of IEVs to the cell surface [36,37]. The A36 protein is restricted to the outer IEV envelope and when this envelope fuses with the cell membrane A36 remains localised on the plasma membrane beneath the CEV particle [38] where it induces nucleation of actin polymerisation through the Arp2/3 complex [11,39]. A36 is the only IEV-associated protein reported to interact with the kinesin-1 complex [40], although this has only been demonstrated with over-expressed A36 protein fragments and not with wild-type A36 protein at endogenous levels during infection. Loss of A36 does not prevent transport of IEV on microtubules to the cell periphery although the efficiency is reduced [41] suggesting there are other proteins involved. A36 possesses a bipartite tryptophan-acidic residue (WD/WE) kinesin binding motif [37,42] through which it interacts with the kinesin-1 complex and without which IEV egress is reduced [37,43]. The F12 protein also possesses a single WD/WE type motif critical for its function [37] and shows some similarity to KLCs (though a recent report has suggested that F12 is more closely related to DNA polymerases of bacteriophage origin [44]). To date, however, no interaction between F12 and the kinesin-1 complex has been described. There is evidence that F12 interacts with A36 [45] and E2 [46]. Deletion of either F12 or E2 results in a smaller plaque phenotype and a severe reduction in EEV/CEV formation [47,48]. Although there has been some suggestion that F12 and E2 deletion viruses show a defect in the IEV wrapping process [46,48], fully formed IEV/CEV particles have been documented and quantified to similar levels as wild-type viruses [36,37,48]. Both F12 and E2 remain associated with IEVs during their egress to the cell periphery but dissociate from IEVs prior to virion release at the cell surface [46].

In this report, evidence of an interaction between F12 and the kinesin-1 complex is presented. However, this interaction requires the presence of E2, not A36. Unlike A36, which binds both KLC1 and KLC2, the F12/E2 complex shows a distinct preference for KLC2 association. This preference is due to F12/E2 binding the KLC2 C-terminus, a region with considerable variability between different isoforms. Lastly, siRNA knockdown of KLC1, but not KLC2, results in more efficient transport of IEV particles to the cell surface and a larger plaque phenotype. In contrast, knockdown of KLC2 gave no such alteration, but knockdown of KLC2 and KLC1 simultaneously removed the plaque size enhancement deriving from knockdown of only KLC1. These results suggest redundancy and a potential complex interplay between different KLC isoforms used for VACV transport.

## Results

### F12 co-immunoprecipitates with kinesin light chain

The VACV F12 protein is associated with IEV particles [36] and has some similarity to KLCs and has a WD/E kinesin binding motif [37], suggesting F12 might form part of the kinesin complex associated with IEV particles. However, attempts to co-precipitate endogenous components of the kinesin-1 complex with an epitope-tagged F12 protein from HeLa or HEK 293T cells infected with a virus expressing a C-terminal HA-tagged F12 protein (vF12-HA) were unsuccessful. Therefore, the experiments were repeated using ectopic overexpression of epitope-tagged components of kinesin-1.

**Fig 1:**
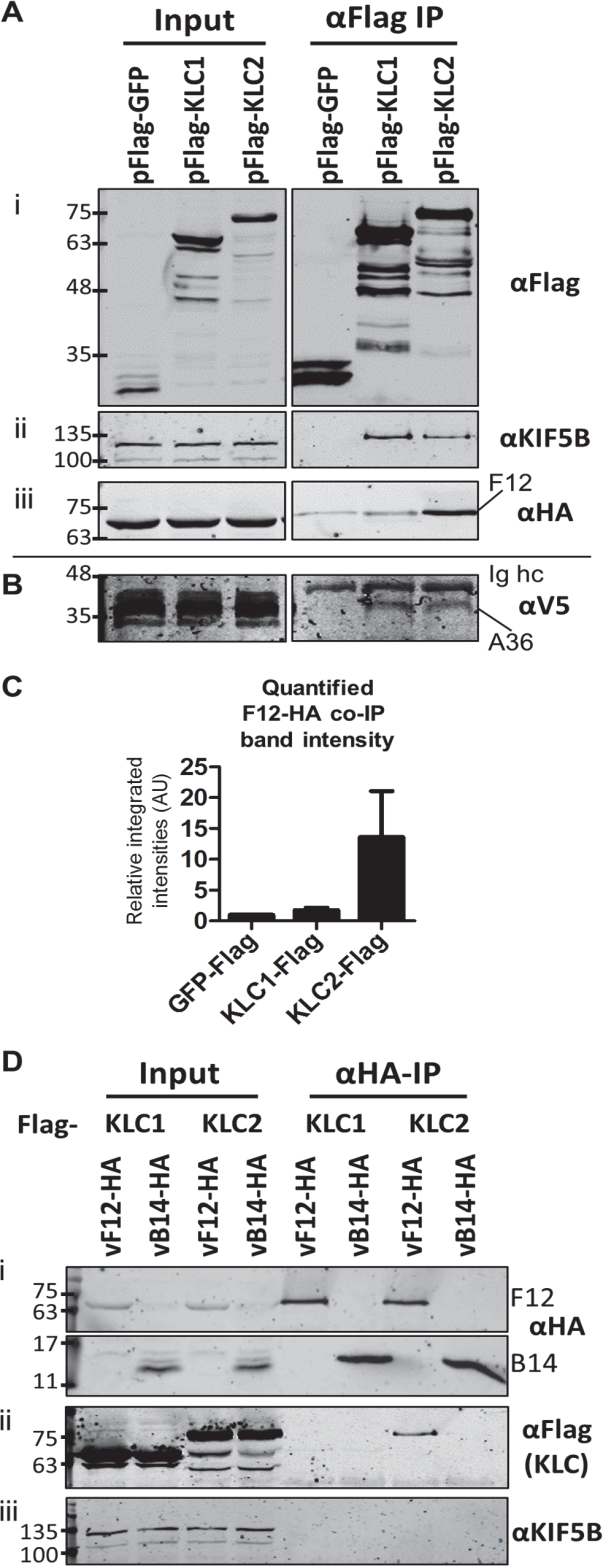
F12 co-immunoprecipitates with kinesin light chain isoform 2. (A) SDS-PAGE and immunoblot analysis of anti-Flag immunoprecipitations. HeLa cells were transfected with plasmids expressing Flag-tagged GFP, KLC1 or KLC2 and were infected 24 h later with vF12-HA (5 PFU/cell) for 14 h. Cell lysates were prepared and immunoprecipitated with anti-Flag antibody. (i) Clarified cell lysate (Input) and immunoprecipitated samples were immunoblotted with an anti-Flag antibody. (ii) As in (i) but immunoblotted with anti-KIF5B to show equal loading of cell lysate (Input) and the ability of Flag-KLC1 and Flag-KLC2 to associate with the endogenous kinesin-1 complex (αFlag IP). (iii) As in (i) but immunoblotted with an anti-HA antibody. (B) The experiment described in (A) was repeated in HeLa cells expressing a V5 epitope-tagged A36 protein. Samples were immunoblotted with anti-V5 antibody. (C) The experiment shown in (A) (iii) was repeated in triplicate and band intensities of co-immunoprecipitated F12 were quantified using a LiCor Odyssey Infrared Imager. Numbers represent the relative integrated intensities (with local background correction) normalised to the intensity of the band in the pFlag-GFP lane of 3 independent experiments ±sd. (D) SDS-PAGE and immunoblot analysis of a reciprocal anti-HA immunoprecipitation. HeLa cells were transfected with plasmids expressing either Flag-tagged KLC1 or KLC2 and were infected 24 h later with vF12-HA or vB14-HA. HA-tagged proteins were immunoprecipitated using anti-HA antibody-coated beads. Samples were immunoblotted with (i) anti-HA, (ii) anti-Flag and (iii) anti-KIF5B (input loading control) antibodies. The positions of molecular mass markers (kDa) are shown on the left for all immunoblots.

HeLa cells were transfected with plasmids expressing N-terminal Flag-tagged murine alleles of KLC1 and KLC2 and were infected subsequently with a virus expressing the F12 protein fused to an HA-epitope tag (vF12-HA) [36]. Flag-tagged proteins were immunoprecipitated using anti-Flag antibody-conjugated agarose beads (Fig. 1A i). Samples were immunoblotted for endogenous Kif5B (kinesin heavy chain) as a cell lysate loading control (Fig. 1A ii). Co-precipitation of endogenous Kif5B with ectopically expressed murine KLC1 and KLC2, but not with GFP indicated that both murine KLC isoforms are able to interact with the human kinesin-1 complex. This is consistent with murine KLC1 and KLC2 sharing 98–99% amino acid similarity with their human counterparts (see supplemental information S1 Fig.). HA-tagged F12 co-precipitated with KLC2 and to a much lesser extent with KLC1 (Fig. 1A iii). Quantitation of F12-HA band intensities showed that F12 co-precipitated with KLC1 only slightly above background (with Flag-GFP), while the co-precipitation with KLC2 was higher (Fig. 1C).

A36 was reported to interact with both KLC1 and KLC2 [43]. To verify that the Flag-tagged KLC isoforms interact with cargo proteins and that the preference of F12 for KLC2 was a genuine property of F12 and not of the KLC alleles being used, the ability of A36 to co-immunoprecipitate with these Flag-tagged KLC isoforms was tested. However, using an anti-A36 monoclonal antibody an interaction between A36 and either KLC isoform during virus infection was not detected. Therefore, the experiment was repeated using a HeLa cell line expressing A36 tagged with a V5 epitope (V5-A36) [15]. Under these conditions V5-A36 co-precipitated with both Flag-KLC1 and Flag-KLC2 (Fig. 1B), consistent with previous reports that A36 binds KLC isoforms 1 and 2 when over-expressed [43].

The F12-KLC2 interaction was investigated with a reciprocal immunoprecipitation. HeLa cells transfected with either Flag-KLC1 or Flag-KLC2 were infected with vF12-HA, or with a VACV expressing HA-tagged B14, a cytoplasmic VACV protein that contributes to virulence [49] and blocks NF-κB activation by binding to IKKβ [50]. HA-tagged proteins were immunoprecipitated and KLC2, but not KLC1, co-precipitated with F12-HA and not with B14-HA (Fig. 1D ii). Blotting for endogenous Kif5B failed to detect any KHC co-precipitating with F12 (Fig. 1D iii), suggesting that F12 interacts with the kinesin-1 complex through an interaction with KLC.

### Subcellular distribution of ectopically-expressed epitope-tagged KLC mirrors that of its endogenous counterpart

**Fig 2:**
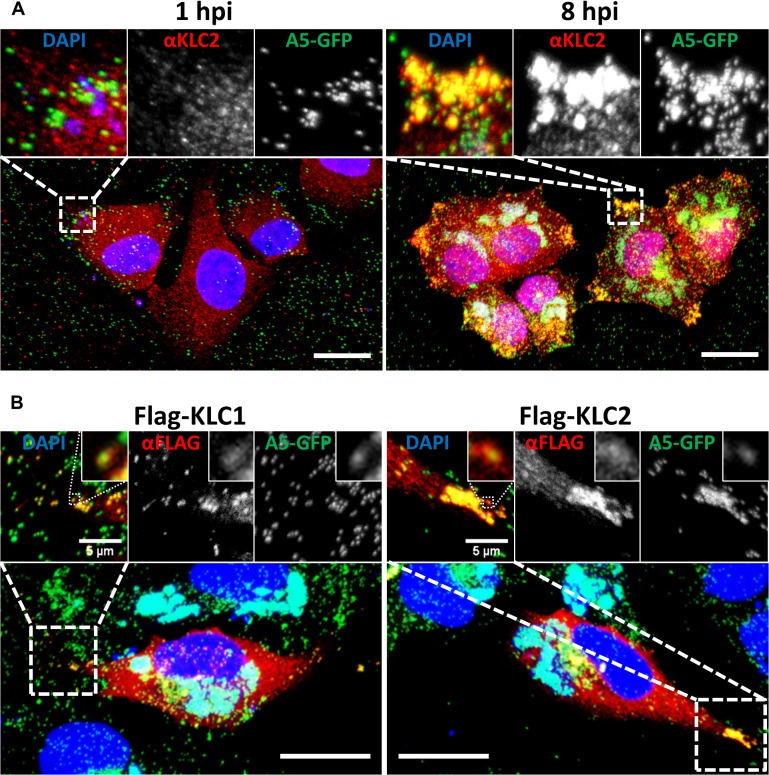
KLC1 and KLC2 associate with peripheral virions. Confocal laser scanning microscopy of HeLa cells grown on glass coverslips. (A) Cells were infected with vA5GFP at 5 PFU/cell and fixed at 1 or 8 hpi. Cells were immunostained with anti-KLC2 antibody (red) and mounted in Mowiol-containing DAPI to stain DNA (blue). (B) HeLa cells were transfected with Flag-KLC1 or Flag-KLC2 and infected with vA5GFP at 5 PFU/cell, fixed 8 hpi, immunostained with an anti-Flag antibody (red) and mounted in Mowiol containing DAPI (blue). Images shown are maximum intensity projections of z-stack data sets acquired of the complete cell volume. The boxed regions are expanded in the inserts. Scale bars represent 20 μm, or when indicated, 5 μm (inserts).

Immunofluorescence and electron microscopy have shown that endogenous KLC associates with IEV particles trafficking to and at the cell periphery [37]. These studies used either the mouse monoclonal 63–90, a pan-specific antibody that recognises an epitope present on all KLC isoforms [51], by immunoelectron microscopy, or the L2 mouse monoclonal that recognises only KLC1 for immunofluorescence. To confirm that endogenous KLC2 associates with virions, HeLa cells were infected with vA5L-GFP [20] and analysed by confocal microscopy using an antibody specific for KLC2 (Fig. 2A). The KLC2 distribution matches the distribution of KLC1 described previously [37]. At late times post infection (pi) KLC2 is highly enriched at peripheral accumulations of GFP-positive virions that have trafficked to the cell periphery (compare 1 hpi with 8 hpi, Fig. 2A inserts).

Fig. 1 showed that murine KLC isoforms expressed from plasmids [52] interacted with endogenous human KHC and they each co-precipitated the A36 protein to similar levels. Consistent with these proteins being functional, immunofluorescence confocal microscopy showed that in HeLa cells infected with vA5L-GFP both Flag-KLC1 and Flag-KLC2 co-localised with GFP-positive virions at the cell periphery late (8 h) during infection, similar to their endogenous counterparts (Fig. 2B, inserts).

### Infection is required for the F12-KLC2 interaction

**Fig 3:**
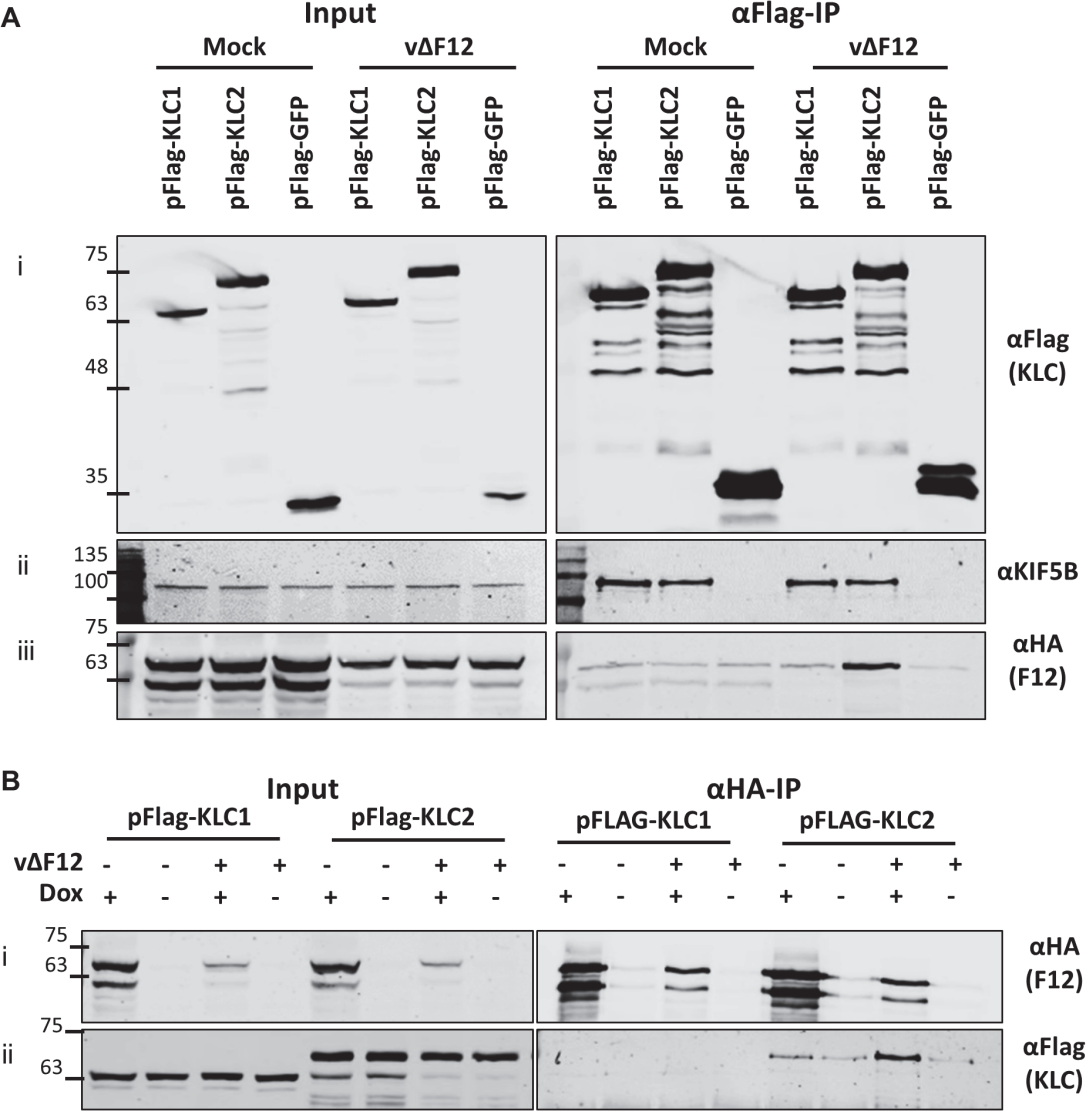
The F12/KLC2 interaction requires virus infection. SDS-PAGE and immunoblot analysis of reciprocal F12-HA and Flag-KLC co-immunoprecipitations in the presence and absence of infection. Clarified cell lysates were generated from HEK TRex-F12-HAco cells transfected with either pFlag-KLC1, pFlag-KLC2 or pFlag-GFP and infected 24 h later with vΔF12 or mock-infected for 14 h as indicated. F12-HA expression was induced by addition of doxycycline for all samples in (A) and as indicated (dox +) in (B). Cell lysates were subjected to either anti-Flag IP (A) or anti-HA IP (B) and the levels of co-immunoprecipitated proteins were analysed as in Fig. 1. The positions of molecular mass standards (kDa) are shown on the left.

The association of F12 and KLC2 might be direct or require another factor. This was tested by ectopic expression of F12 using a HEK293T cell line expressing codon-optimised F12-HA (HEK TRex-F12co-HA) inducibly upon addition of doxycycline (Dox, see Materials and Methods). These cells were transfected with plasmids expressing Flag-KLC1, Flag-KLC2 or Flag-GFP and 24 h later were infected with vΔF12 [47], followed by addition of doxycycline to induce F12-HA expression. As before, F12-HA co-precipitated with KLC2 during virus infection (Fig. 3A iii), but only background levels were detected with KLC1. This confirmed the interactions seen in Fig. 1 using a different cell type. Notably, both KLC1 and KLC2 did not interact with F12-HA above background levels in mock-infected samples (Fig. 3A iii), suggesting that an additional VACV protein or VACV-induced cellular factor was required. In the reciprocal immunoprecipitation using α-HA, KLC1 co-precipitation was not detected either in the presence or absence of virus infection, while KLC2 co-precipitation was detectable clearly in the presence of virus infection (Fig. 3B ii). In the absence of VACV infection KLC2 co-precipitated with F12 at levels only marginally above background (Fig. 3B ii). This may be a genuine low level interaction, or may represent increased non-specific association of KLC2 when higher levels of F12-HA are made in uninfected cells.

### VACV protein E2 is required for F12-KLC2 co-precipitation

**Fig 4:**
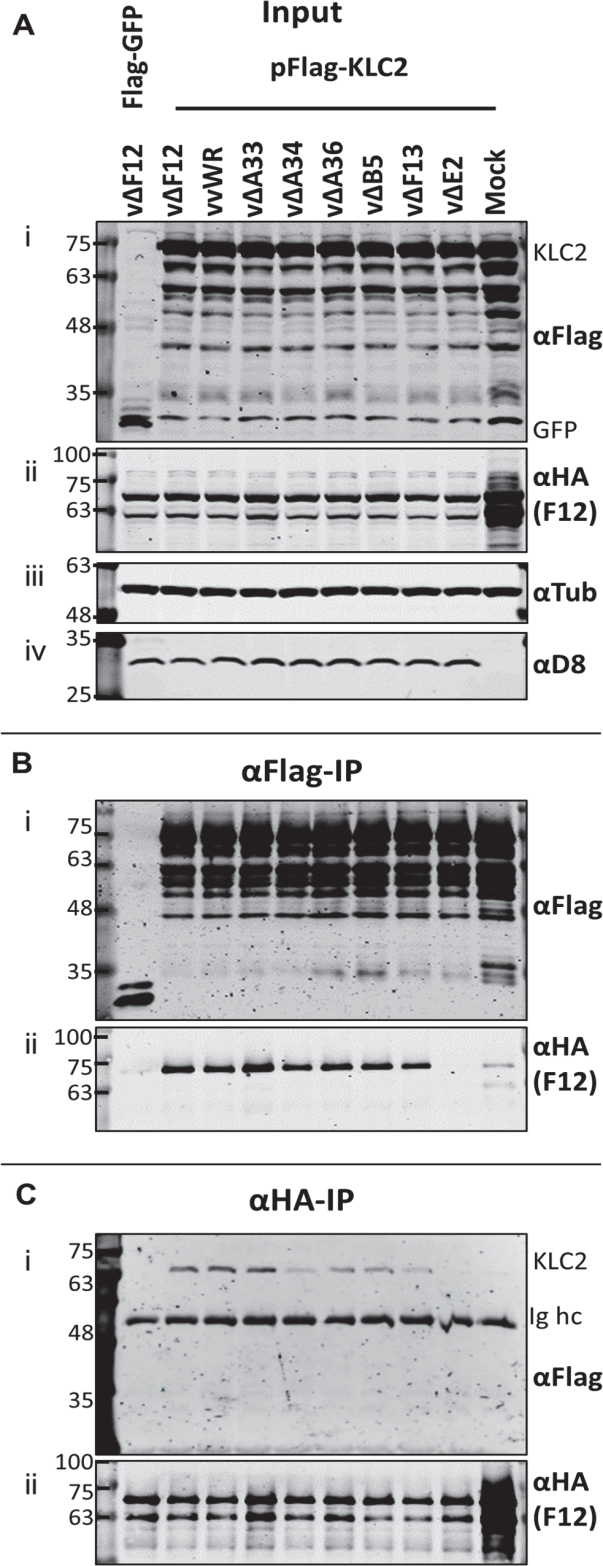
The F12/KLC2 interaction requires expression of E2. SDS-PAGE and immunoblot analysis of F12/KLC2 co-immunoprecipitations carried out in the presence of infection with a panel mutant VACVs lacking individual IEV proteins. Clarified cell lysates were generated from HEK TRex-F12-HAco cells that had been transfected with pFlag-KLC2 and 24 h later infected with VACV WR (vvWR), vΔF12, vΔA33, vΔA34, vΔA36, vΔB5, vΔF13, vΔE2 or mock-infected. These cells had also been induced to express F12-HA by addition of doxycycline (+Dox). One sample was transfected with pFlag-GFP as a negative control to measure background levels of co-immunoprecipitated F12-HA. (A) Input cell lysate samples were immunoblotted for Flag (i, KLC), HA (ii, F12), tubulin (iii, loading control) and D8 (iv, infection level control). Cell lysates were subjected to anti-Flag IP (B) and anti-HA IP (C), and co-precipitating proteins were analysed by blotting for Flag (i) and HA (ii). The positions of molecular mass standards (kDa) are shown on the left.

Given that F12-KLC2 co-precipitation was seen only during VACV infection, it is possible that one or more VACV proteins are required for this interaction, and the proteins present associated with the outer membrane of the IEV particle are the most likely candidates. These include A33, A34, A36, A56, B5 and E2, and of these, A36 [45] and E2 [46] were reported to interact with F12. To investigate if particular IEV outer membrane proteins were involved in the F12-KLC interaction, a panel of VACV mutants lacking A33 [53], A34 [1], A36 [54], B5 [55], F13 [34] or E2 [48] were used to infect the F12-HAco-expressing cell line that had been transfected with pFlag-KLC2. Cell lysates were immunoprecipitated with an anti-Flag (Fig. 4B) or anti-HA (Fig. 4C) antibody and samples were analysed by immunoblotting. Using either antibody, the F12-KLC2 association was detected in samples transfected with Flag-KLC2 and infected with vΔF12. The panel of deletion viruses used in this experiment all express untagged F12, the HA-tagged F12 being produced by the host cell. To determine if the presence of untagged F12 interferes with the F12-HA/Flag-KLC2 interaction, a control was included in which the cells were infected with wild-type VACV WR (vvWR), expressing untagged F12, in conjunction with F12-HA expressed by the cell line. No significant difference in the levels of F12-HA/KLC2 co-precipitation were detected in the presence or absence of untagged F12 (compare the second and third lanes from left). The input samples were blotted for tubulin (Fig. 4A iii) to control for the protein levels used in the immunoprecipitation and for the VACV protein D8 (Fig. 4A iv) to control for equal infection. The F12-HA/KLC2 association was maintained in cells infected by all of the viruses tested except vΔE2 (Fig. 4B and Fig. 4C), suggesting a critical requirement for E2 in the F12/KLC2 interaction.

### E2 is necessary and sufficient to mediate the F12/KLC2 interaction

**Fig 5:**
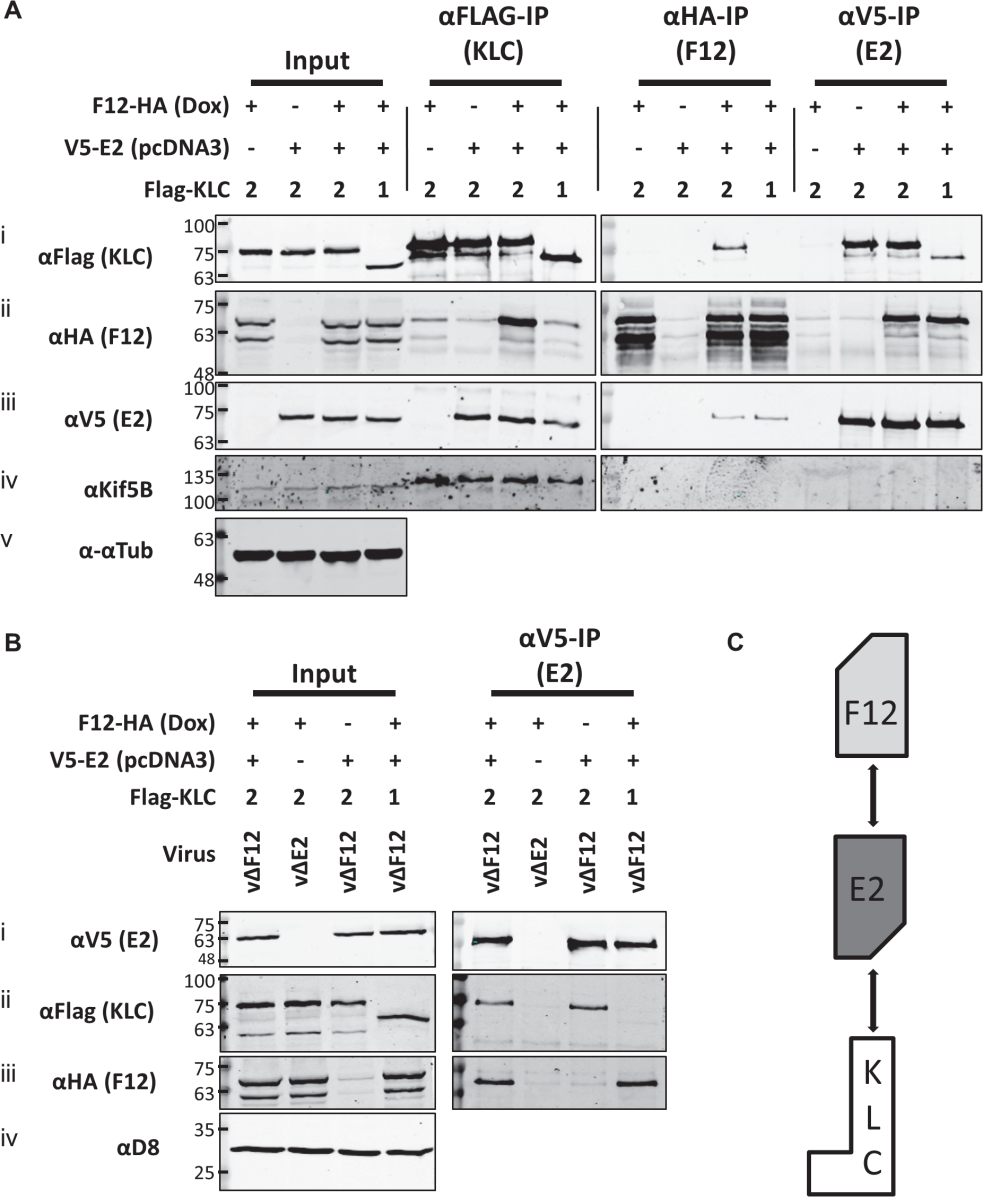
E2 interacts with KLC2 and is necessary and sufficient to mediate F12 interaction with KLC2. (A) SDS-PAGE and immunoblot analysis of KLC (αFlag-IP), F12 (αHA-IP) and E2 (αV5-IP) immunoprecipitations carried out in parallel on cell lysates generated from HEK TRex-F12-HAco expressing Flag-KLC (isoform 1 or 2 as indicated), and expressing F12-HA (induced by addition of doxycycline) and/or V5-E2 (by transfection of pcDNA3-V5-E2co) as indicated. Clarified cell lysates (Input) and immunoprecipitated samples were immunoblotted with αFlag, αHA, αV5, αKif5B and α-αtubulin. (B) The E2 immunoprecipitation (αV5-IP) was repeated in the presence of virus infection to confirm the KLC isoform specificity. In addition to ectopic expression of Flag-KLC, F12-HA and V5-E2 cells were infected at 5 PFU/cell with either vΔF12 or vΔE2 (as indicated) such that samples only expressed either F12 or E2 or both proteins. Clarified cell lysate samples (Input) were immunoblotted for VACV protein D8 (αD8) to control for equal infection levels. The positions of molecular mass standards (kDa) are shown on the left. (C) Model for the order of interaction of F12, E2 and KLC.

To test if E2 was the only VACV protein required for F12 to associate with KLC2, plasmids expressing epitope-tagged codon-optimised E2 (E2co) carrying either an N-terminal V5-tag or HA-tag, driven by a human cytomegalovirus promoter were constructed (see Materials and Methods). HEK TRex-F12-HAco cells transfected with plasmids expressing Flag-KLC1 or 2 were co-transfected with the V5-E2co expressing plasmid or empty vector control in the absence of virus infection (Fig. 5A). F12-HA expression was induced by treating with doxycycline. Lysates were immunoprecipitated with α-Flag, α-HA and α-V5. Pull-down of Flag-KLC2 (Fig. 5A i) co-precipitated F12-HA only when E2 was present (Fig. 5A ii). However, V5-E2 co-precipitated with Flag-KLC2 whether or not F12 was present (Fig. 5A iii), indicating that F12 is not required for E2 to interact with the kinesin-1 complex. The published F12/E2 interaction [46] was confirmed by the observation that F12 co-precipitated with E2 (Fig. 5A ii) and this interaction was maintained in the reciprocal IP (Fig. 5A iii). The levels of F12-E2 co-precipitation did not differ when KLC1 or 2 was over-expressed. KLC1 did not co-precipitate with F12 to detectable levels in reciprocal IPs (Fig. 5A i and Fig. 5A ii) but some co-precipitation was observed for KLC1 and E2 (Fig. 5A i and Fig. 5A iii), although levels were much lower than observed with KLC2. Repeating the V5-E2 IP in infected cells (Fig. 5B) confirmed the observations using uninfected cells, except that the E2/KLC interaction showed a higher specificity for KLC2 in infected cells (Fig. 5B ii). Taken together, these data show that E2 interacts with KLC2 and F12 interacts with E2 to form the F12/E2/KLC2 complex (Fig. 5C).

**Fig 6:**
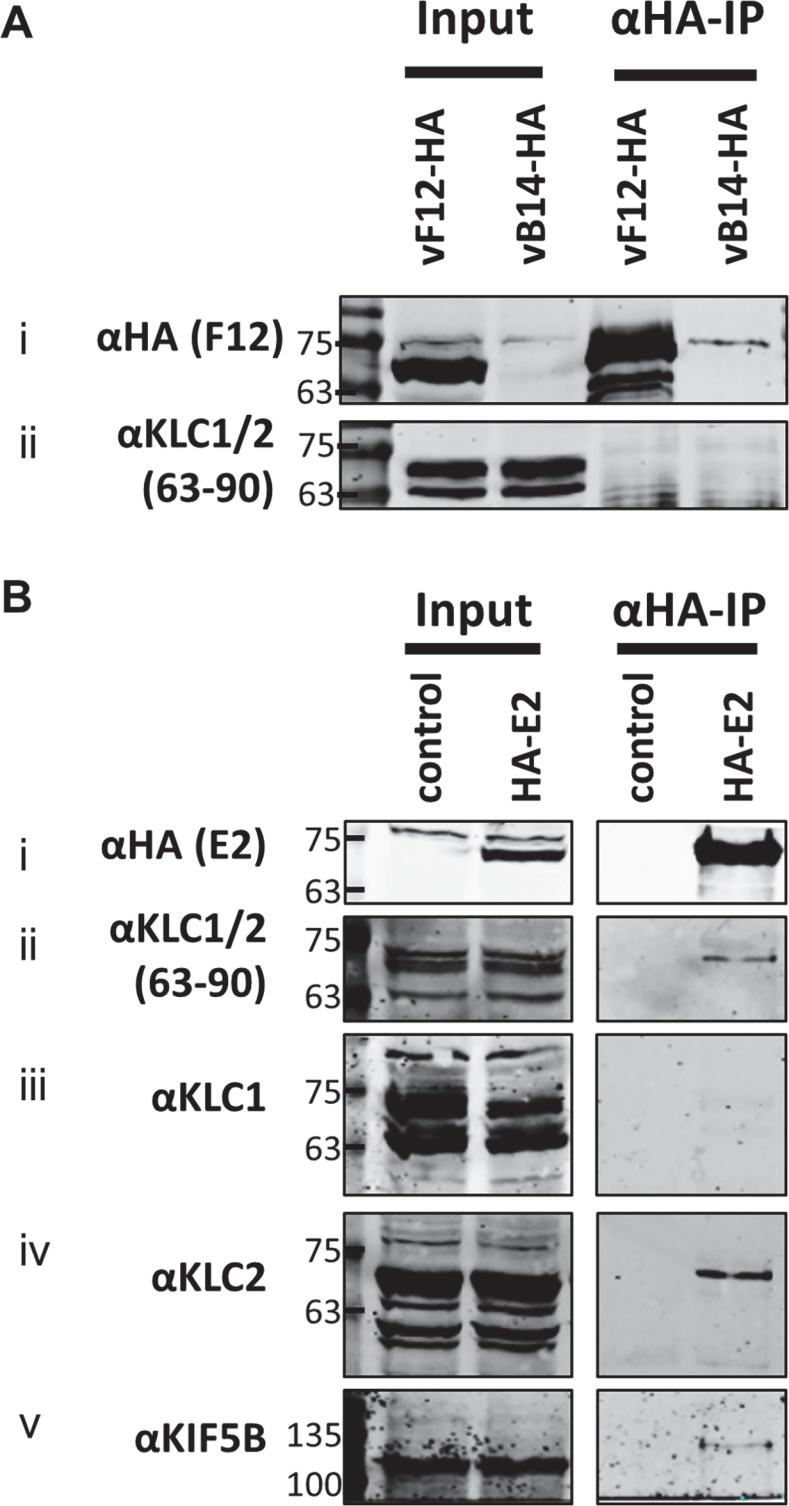
Endogenous KLC2 co-immunoprecipitates with E2. (A) SDS-PAGE and immunoblot analysis of α-HA-IP from HEK 293T cells infected with either vF12-HA or vB14-HA at 5 PFU/cell and harvested 14 hpi. Clarified cell lysates (Input) and α-HA immunoprecipitated samples were immunoblotted with the antibodies indicated on the left of the figure. (B) SDS-PAGE and immunoblot analysis of α-HA immunoprecipitation from lysates generated from HEK 293T cells transfected with a plasmid encoding HA-tagged E2 or a control plasmid as indicated. Samples were probed for the precipitated E2 protein (i) and for co-precipitation of KLC using the 63–90 antibody (ii). The co-precipitating KLC isoform identity was confirmed by immunoblotting with antibodies specific for KLC1 (iii) and KLC2 (iv). Co-precipitation of the entire kinesin-1 complex with E2 was confirmed by immunoblotting with the α-Kif5B antibody (v).

As mentioned, attempts to precipitate endogenous KLC with F12-HA were unsuccessful. This was also true using larger amounts of cell lysate, larger volumes of anti-HA antibody-coated beads and longer incubation times (Fig. 6A). However, because the results shown in Fig. 4 and Fig. 5 indicated that E2 can interact with KLC2 without F12, interactions between endogenous kinesin-1 (KLC and KHC) and epitope-tagged E2 were investigated. The HA-tagged E2co plasmid was transfected into 293T cells, E2co was precipitated with anti-HA beads and samples were analysed by immunoblotting using the 63–90 antibody. This detected several bands in the input lanes (Fig. 6B ii) corresponding to the different KLC isoforms present (possibly including variants of KLC1 produced by differential splicing [56]). Notably, a band corresponding to one of the larger isoforms was detected co-precipitating with HA-E2 but not in the negative control (Fig. 6B ii). In the literature this upper band is often assumed to correspond to KLC2 with the lower band corresponding to KLC1 [31,51]. To confirm that the co-precipitated protein was KLC2, immunoblotted membranes were stripped and re-probed with antibodies specific for KLC1 (Fig. 6B iii) and KLC2 (Fig. 6B iv). Only the anti-KLC2 antibody recognised the band co-precipitating with E2, although the entire kinesin-1 complex co-precipitated with E2 because KHC was also detected (Fig. 6B v). These results indicate that E2 interacts with endogenous human KLC2 but not KLC1 and this specificity is indistinguishable from the murine KLCs expressed ectopically. This retention of this specificity between the human and murine proteins is consistent with the very high amino acid conservation of these proteins (S1 Fig.).

### Measuring the effect of KLC1 vs KLC2 siRNA knockdown on virus egress

**Fig 7:**
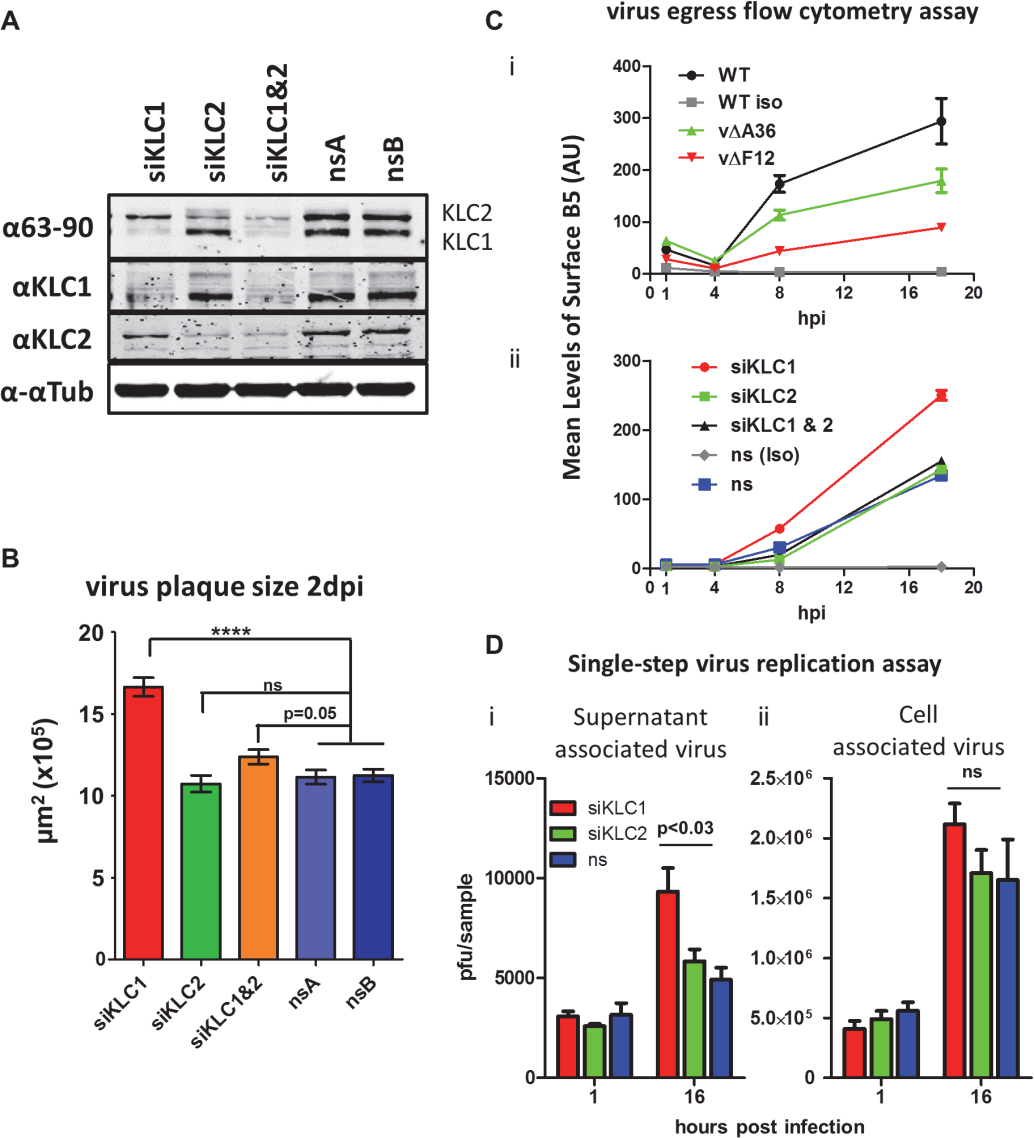
The effect of KLC knock-down by siRNA on virus egress. (A) SDS-PAGE and immunoblotting analysis of the efficiency of siRNA knockdown of KLC1 and KLC2 in the human osteosarcoma cell line U-2 OS. Cells were treated with siRNA targeting KLC1 (siKLC1), or KLC2 (siKLC2) or a mix of both (siKLC1 & 2) and compared to cells treated with two independent non-targeting siRNAs (nsA and nsB). Cells were harvested 72 hpi and protein levels were analysed by SDS-PAGE. Tubulin levels were measured to confirm equivalent protein loading levels using an antibody specific to α-tubulin. Levels of KLC1 and KLC2 were measured by staining both with the pan-KLC 63–90 antibody, detecting both KLC1 (lower band) and KLC2 (upper band), and antibodies specific for KLC1 and KLC2. (B) Plaque size determination of vA5GFP on siRNA treated U-2 OS cells. Cells were treated with siRNA to KLC1, KLC2, KLC1&2 or two independent non-silencing RNAs (nsA and nsB). Monolayers of siRNA-treated cells were infected with vA5GFP to generate well separated plaques by 3 dpi. Cells were fixed and plaques positive for GFP expression were imaged using an inverted fluorescence microscope with a mounted digital camera and plaque surface area was measured using Axiovision (Zeiss) software. The average size of 20–35 plaques per sample and 3 replicate samples per condition were calculated and compared by student t-test (**** p<0.0001). (C) Estimation of virus egress from siRNA-treated cells by flow cytometry. Cells infected with vA5GFP at 5 PFU/cell and stained at various times pi prior to fixation for the CEV-associated B5 protein. Levels of staining were quantified by flow cytometry. (i) To validate this method of measuring egress an initial experiment compared three viruses known to display different levels of virion egress; vA5GFP (WT), vA5GFP-ΔA36 (vΔA36) and vA5GFP-ΔF12 (vΔF12). Background staining levels were monitored by including a sample stained with an isotype control antibody (iso). The three viruses showed levels of surface staining similar to their known relative levels of virion egress. (ii) To measure the effect of siRNA treatment on egress, cells were treated with siRNAs for 48 h and then infected with vA5GFP and stained for surface B5 at the indicated times. (D) Single step growth curve of released and cell-associated virus from siRNA-treated cells. U-2 OS cells were treated with siRNA targeting either KLC1 or KLC2 or a non-silencing (ns) control RNA and infected with vA5GFP at 10 PFU/cell 48 h after siRNA treatment. The supernatant (i) and cells (ii) were harvested separately at 1 hpi and 16 hpi. The infectious virus titre of triplicate samples was determined by plaque assay and numbers were analysed by student’s T-test.

To dissect the relative contribution of KLC1 and KLC2 to IEV trafficking, the effect on virus egress and plaque size of siRNA-mediated knockdown of the two KLC isoforms 1 and 2 was investigated. Knockdown of KLC1, KLC2 or both was achieved using a mixture of several siRNAs (Fig. 7A). Infection of cells in which KLC2 was knocked down had no discernible effect on plaque size (Fig. 7B) or virus egress to the cell surface as measured by surface B5 staining and flow cytometry (Fig. 7C), despite the F12/E2 complex interacting with KLC2. In contrast, siRNA knockdown of KLC1 caused increased virus egress (Fig. 7C) and plaque size (Fig. 7B). Interestingly, although knockdown of KLC2 had no effect alone, if KLC1 and KLC2 were knocked down together, the increased virus egress and plaque size deriving from loss of KLC1 was abrogated (Fig. 7B and C). To check that alterations in virus egress and plaque size following siRNA treatment were not just a consequence of alterations in virus replication, the virus associated with cells and in the culture medium were measured (Fig. 7D). This showed that there was no significant difference in titres of infectious virus within or attached to cells (Fig. 7D i), but there was a small but significant increase in virus released from the cells after knockdown of siRNA (Fig. 7D ii). This is consistent with knockdown of KLC1 enhancing virus egress to the cell surface.

### Mapping the F12/E2 interaction with KLC2

**Fig 8:**
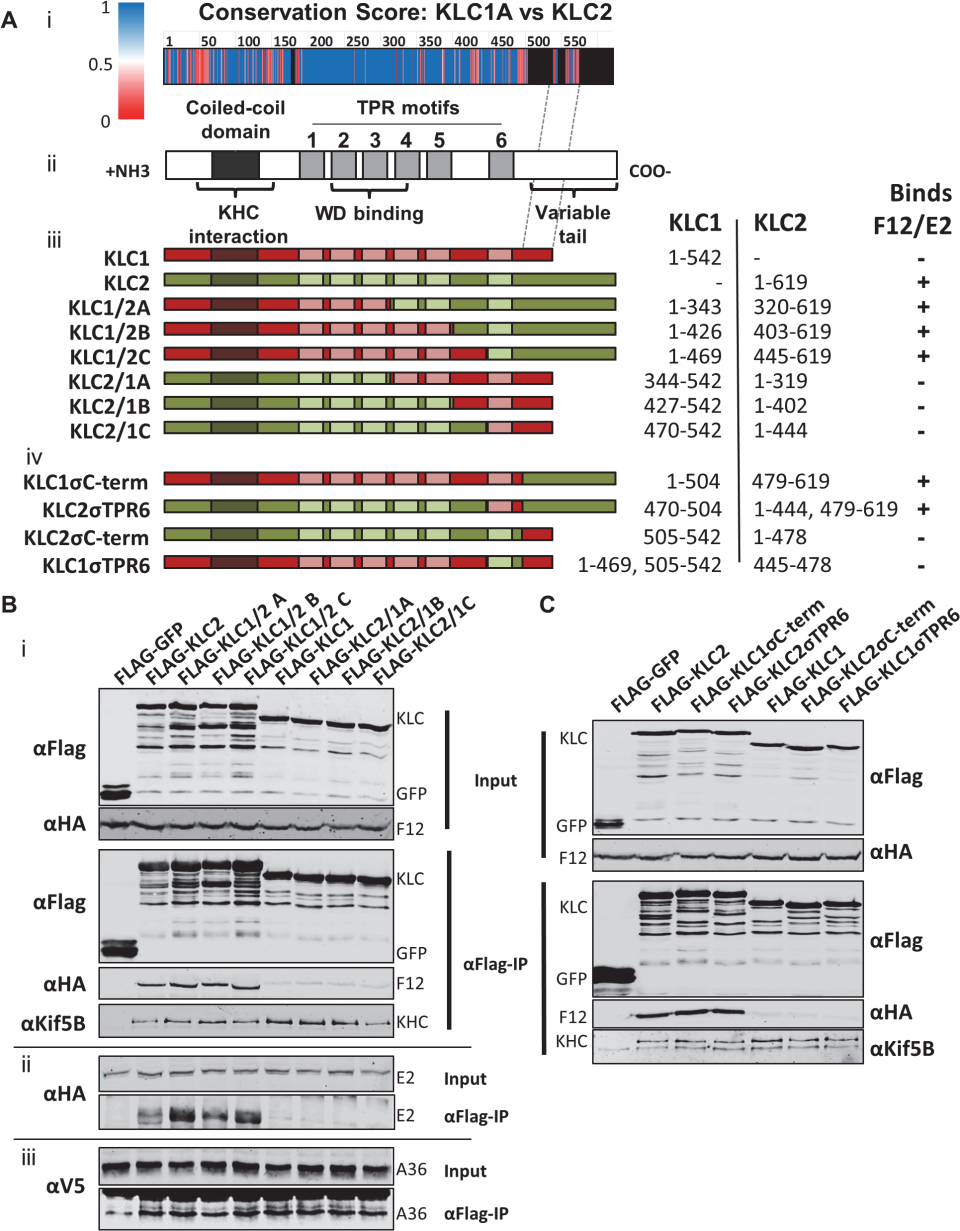
The F12/E2 interaction with KLC maps to the KLC2 C-terminal tail. (A) Schematic representation of KLC and chimeric alleles used. (i) Conservation score plot of murine KLC1 and KLC2 protein sequence alignment (shown in supplemental information S2 Fig.). The physiochemical conservation was calculated for each residue using the PET91 matrix (1 = complete conservation, 0 = no conservation, see key for colour values, positions within KLC2 that have no corresponding KLC1 residue are coloured black) and plotted onto the KLC domain organisation diagram to scale (note the alignment of the KLC1 C-terminal tail to a region in the centre of the KLC2 C-terminal tail as denoted by hatched grey lines). (ii) KLC domain organisation. All KLC molecules possess an N-terminal coiled-coil region that mediates interaction with KHC, a TPR region consisting of 6 copies of the tetratricopeptide repeat (TPR) motif, that affects interaction with certain cargo proteins containing a tryptophan acidic (WD) motif, and a highly variable C-terminal tail. (iii) Schematic of KLC1/KLC2 chimeric proteins generated. The regions used in each chimera are colour coded; red for KLC1 and green for KLC2. The amino acid positions of KLC1 and KLC2 included in each chimera are listed alongside. A summary of the results described in results below is also shown. (B) and (C) Co-immunoprecipitation analysis of the interaction of KLC chimeras with the F12/E2 complex. (i) Cells transfected with FLAG-KLC chimeras were infected with vF12-HA and clarified cell lysates produced 16 hpi. Chimeric Flag-KLC proteins were immunoprecipitated and co-precipitating F12-HA and KHC were analysed by immunoblot as described for Fig. 1A. (ii) The experiment was repeated using vHA-E2 (a virus expressing HA-tagged E2) to analyse the interaction of E2 with the various chimeras. (iii) The experiment was repeated in a HeLa cell line expressing V5-tagged A36 to analyse the ability of A36 to bind to the different chimeras. (B) shows results for the chimeras described in (a iii) and (C) shows the more detailed mapping using the chimeras described in (a iv).

All KLC isoforms are made up of an N-terminal coiled-coil region that mediates KHC association, and a C-terminal TPR motif-containing region that mediates interaction with cargo proteins [57] (Fig. 8A ii). Sequence alignment of murine KLC1 (spliceform A, accession number NM_008450.2) and KLC2 (accession number NM_008451.2) shows a high level of amino acid identity, particularly in the TPR region. Clusters of sequence variation occur within TPR motifs 4, 5 and 6, the linker region between TPR 5 and 6, and in particular the C-terminal tail region (Fig. 8A i), which could contribute to the selective binding of cargo proteins (such as E2) with a particular KLC isoform.

To map the site of interaction between KLC and F12/E2, chimeric KLCs were generated in which regions of KLC1 and KLC2 were switched (Fig. 8A iii), focusing on the TPR domain that showed the lowest conservation scores (Fig. 8A i). Immunoprecipiation of these Flag-tagged KLC chimeras from cells infected with either vF12-HA (Fig. 8B i) or vE2-HA (Fig. 8B ii) showed that only proteins possessing the KLC2 C-terminal TPR co-precipitated the F12/E2 complex. All the chimeric proteins formed part of the kinesin-1 complex, because they interacted with KHC (Fig. 8B i, bottom panel). They also interacted with A36 (Fig. 8B iii), suggesting each chimera, particularly its TPR domain, remained functional.

Additional KLC1/2 chimeras were constructed to determine the contribution of the C-terminal tail or TPR6 in the KLC interaction with E2 (Fig. 8A iv). The E2/F12 complex only immunoprecipitated with KLCs possessing the isoform 2 C-terminal tail (Fig. 8C) regardless of which TPR 6 was present. These results explain the preference of E2 for KLC2 over KLC1 (spliceform A, which lacks this tail).

## Discussion

The VACV F12 protein is involved in IEV egress via an unknown mechanism. F12 shares similarity with both KLC and the proteins that interact with KLC [37] and is associated with IEVs during microtubule-based movement and then dissociates from IEVs prior to the switch to actin-based motility [46]. Therefore, we investigated whether F12 was itself a kinesin-1-interacting protein. Both ourselves and other labs [46] had been unable to detect F12/kinesin-1 interaction by affinity tag pull-down of F12. However, data presented here showed an interaction when epitope-tagged KLC was expressed ectopically. Interestingly F12, unlike A36, discriminated between different KLC isoforms, associating with KLC2 but not KLC1 and the F12/KLC2 association was only detectable in the presence of virus infection. These observations may explain why a F12/KLC interaction had not been detected previously, because those experiments had used reagents specific for KLC1 or had been done in the absence of virus infection. Using a panel of viruses lacking expression of individual IEV-associated proteins, E2 was identified as critical for the F12-KLC interaction. Furthermore, E2 proved to be both necessary and sufficient to mediate the F12/KLC interaction, even in the absence of virus infection.

RNAi knockdown of either KLC1 or KLC2 failed to demonstrate a critical role for VACV egress or spread. However, KLC1 knock-down enhanced virus egress and plaque size. While KLC1 knock-down did not have a statistically significant effect on total virus replication, a slightly increased amount of released virus was observed (Fig. 7D). Knock-down of KLC2 alone had no discernible effect on virus egress or spread, however knockdown of KLC2 and KLC1 reversed the effect of knocking down KLC1 alone (Fig. 7B and C). Until recently it had been accepted that all kinesin-1 complexes contain homologous pairs of KLCs [58]. However, recent proteomics studies have suggested that complexes with different KLC isoforms also exist [59]. results presented here suggest a possible functional interplay between KLC1 and KLC2.

**Fig 9:**
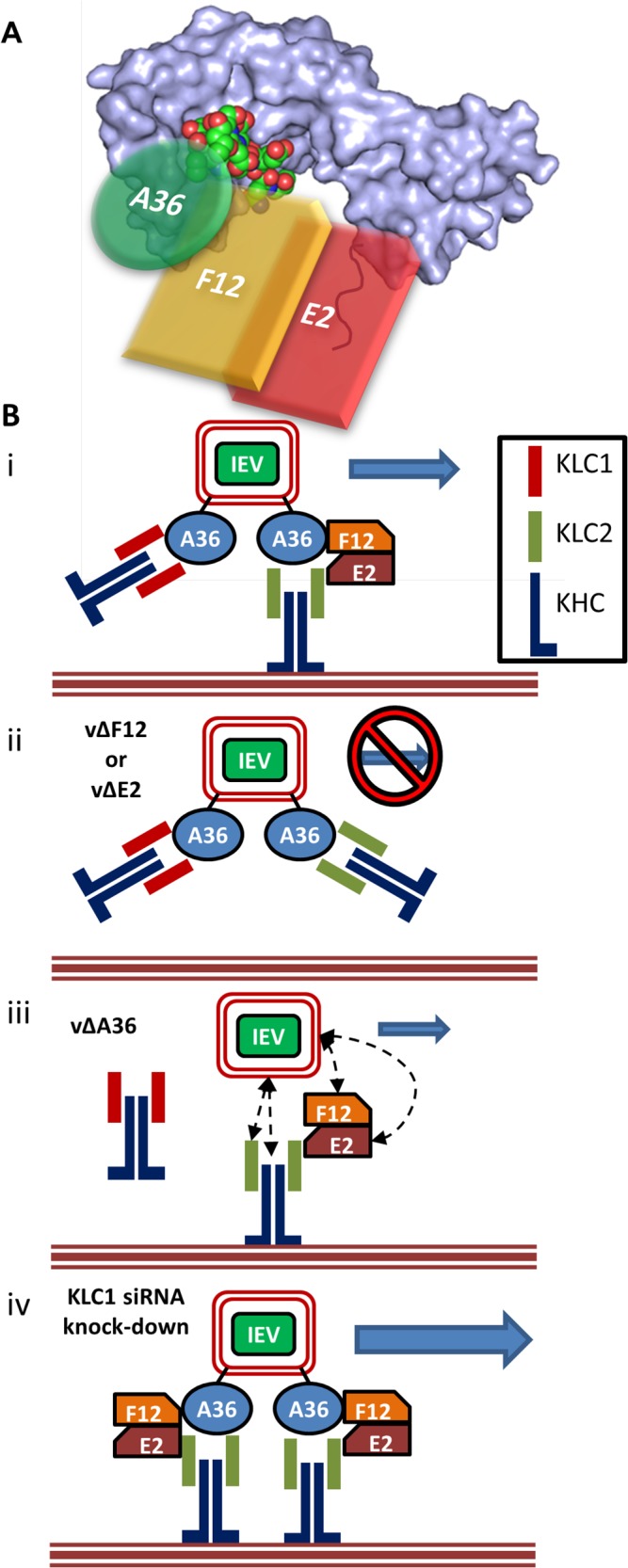
F12 and E2 form part of the IEV trafficking complex. (A) Schematic model (not to scale) of the IEV/kinesin-1 interaction complex showing the potential spacial arrangement of A36, F12 and E2 in relation to the KLC2 TPR structural model (shown as a surface rendering) published by Pernigo *et al* [67] (PDB # 3ZFW). The A36 WD/E motif interacts with the TPR groove in a similar manner to that shown for SifA-kinesin interacting protein (shown as an atomic space filling model) while E2 interacts with the C-terminal tail of KLC. F12 can interact with both A36 and E2. (B) (i) For fully wrapped IEVs to be transported efficiently from the site of wrapping to the cell surface the presence of A36, F12 and E2 is required. (ii) In the absence of either F12 or E2 IEV trafficking is almost entirely abrogated. (iii) In the absence of A36 some IEV egress can still take place. The E2/F12 complex might mediate the interaction between IEVs and kinesin-1 either directly or via another viral or cell protein present in IEVs. (iv) siRNA knockdown of KLC1 may result in an increased association of IEVs with KLC2 containing motor complexes, increasing the efficiency of trafficking.

The interaction of F12/E2 with KLC2 requires the C-terminal tail of KLC2, although binding may also involve other regions of the KLC molecule. The interaction with the C-terminal tail of KLC2 may explain why siRNA knockdown of KLC2 does not block VACV egress for the F12/E2 complex might associate with any KLC isoform that possesses a KLC2-like C-terminal tail. Humans and mice express at least 4 different KLC isoforms, each encoded by a different gene but showing high degrees of sequence similarity. For example, mice and humans KLC1A shares 97% amino acid identity and 99% amino acid similarity, while KLC2 shares 95% identity and 98% similarity, see supplemental information S1 Fig.). KLC1 and KLC2 are expressed most abundantly and ubiquitously, although KLC1 is described as enriched in neuronal cells [31]. KLC3 has been described as specific to developing spermatids [32] and the tissue distribution of KLC4 remains uncharacterised, although expression of both KLC3 and KLC4 has been detected in laboratory cell lines [59]. All isoforms possess a highly conserved KHC-interacting domain, a conserved TPR region (with minor differences as shown in Fig. 9A i) and a highly variable C-terminal tail. The KLC1 locus can produce at least 16 different spliced mRNAs, each encoding a KLC1 differing in the sequence and length of the C-terminal tail [56]. The allele of KLC1 used in this study corresponds to KLC1A, the shortest KLC1 spliceform. Several of the larger KLC1 spliceforms, KLC3 and KLC4 encode C-terminal tails that show strong similarity to KLC2 [56,60]. The larger KLC1 isoforms were detected with the 63–90 and KLC1-specific antibodies after siRNA knockdown of KLC2, but were absent when both KLC1 and KLC2 were knocked down (Fig. 7A lanes 2 and 3). Such KLC1 isoforms, KLC3 and KLC4 may be capable of interacting with F12/E2, suggesting there may be a redundancy in the function of different KLC isoforms in cells and the ability of VACV to utilise them. Different cells express different KLC repertoires and so it may be advantageous for VACV to utilise multiple KLC isoforms to exploit kinesin-based transport in different cell types. However, KLC2, being ubiquitously expressed would be the most attractive target.

It has been proposed that the KLC C-terminal tail plays a role in targeting kinesin-1 complexes to specific subcellular structures, with spliceforms of KLC1 displaying distinct subcellular localisation [61] and differentially affecting the trafficking of different subcellular structures (KLC1B associates with rough endoplasmic reticulum, KLC1D with Golgi [62]). Additionally, the KLC C-terminal tail is the target of a number of post translational modifications that modulate KLC interaction with its binding partners. For example, the C-terminal 25 amino acids of KLC2 contains a motif that can be phosphorylated by casein kinase II, which in turn primes the molecule for phosphorylation by glycogen synthase kinase 3 (GSK3) at multiple serines [63]. This phosphorylation reduces association of kinesin-1 with membrane-bound organelles, suggesting this may be a mechanism of inducing cargo release once at its destination. The putative GSK3 target sequence is conserved between mouse, rat and human KLC2 but not KLC3, KLC4 or many of the characterised KLC1 spliceforms [60]. Two additional serine residues, targeted for phosphorylation by protein kinase A, have also been identified in the KLC2 C-terminal tail (S545 and S582 in human KLC2, S542 and S579 in mouse KLC2 and conserved in KLC3 and KLC4, and several of the KLC1 spliceforms possess one or both). Phosphorylation of both sites is required for the association of the KLC interacting scaffolding protein 14–3–3, which, like F12/E2, shows specificity for KLC2 [60,64].

The role of F12/E2 association with the KLC C-terminal tail remains uncertain. Binding of multiple cargo proteins to different regions of the KLC TPR domain can be cooperative [65]. Structural studies have suggested that association of a WD/E motif (like the ones present in A36 [43]) containing peptide induces a conformational shift in the KLC TPR domain [66,67] and such cargo-induced conformational changes may influence kinesin-1 motor activation. This report is the first confirmation that the F12/E2 complex interacts with the kinesin-1 complex, making it a prime candidate as a regulatory element influencing kinesin-1 activity during egress of IEVs. A36 associates with KLC via interaction of its WD/E motif (shown as an atomic space filling model in Fig. 9A) with the KLC TPR groove (located in the N-terminal half of the TPR domain, Fig. 9A). F12 can bind A36 [45] and E2 [46], though it is unclear if these interactions are cooperative or mutually exclusive, E2 in turn associates with the C-terminal tail of KLC (shown in red, Fig. 9A) forming the IEV trafficking complex. It is possible that the F12/E2 association stabilises or enhances a cargo-induced TPR domain conformational shift. Formation of the trafficking complex is required for IEV kinesin-1 trafficking (Fig. 9B i). F12 and E2 are essential in this complex because viruses lacking expression of either of these proteins produce IEVs that are retained at the site of wrapping [36] (Fig. 9B ii). Viruses lacking F12 or E2 fail to bind kinesin-1 even though A36 is present [37], although we and others found a A36-KLC interaction using over-expressed A36 in the absence of F12 or E2 [40]. Deletion of A36 does not result in a complete loss of IEV egress [41] and so another element connecting IEVs to kinesin-1 must exist (Fig. 9B iii). F12 or E2 might provide the link between kinesin 1 and IEVs either directly or via another IEV protein. Alternatively, the trans-Golgi or early-endosomal membranes used to form IEVs contain cellular membrane proteins, some of which are incorporated into IEVs [68]. Some of these proteins likely affect vesicle trafficking and could form alternative connections between IEVs and the kinesin-1 complex. Both KLC1 and KLC2 associate with trafficking IEVs. If the F12/E2 association is critical for kinesin-1 activation then the association of KLC1 with IEVs might be detrimental to VACV egress, because it would block association with KLC2 and potentially act as a tether. Increased expression of the KLC1E spliceform in the brain has been linked to increased levels of amyloid β plaque formation and the development of Alzheimers disease in mouse models [69], and one model suggested to explain this is that the increased levels of KLC1E interferes with the ability of other KLCs to transport amyloid β precursors correctly [70]. The observation that siRNA-mediated knock-down of KLC1 resulted in enhanced egress suggests there might be a similar interaction between KLC1 and KLC2 during VACV trafficking (Fig. 9B iv). VACV provides a useful tool to enhance our understanding of the cellular roles and properties of the different KLC isoforms and spliceforms.

A36 and F12 each affect IEV egress from the site of wrapping to the cell surface [36,38,40]. The observation that F12 and E2 form a complex and that both proteins are required for the other to associate with IEVs has added E2 to this list [46]. The integral membrane protein A36 is the only reported direct link between IEVs and the kinesin-1 complex, although this has not been shown during infection with wild-type endogenous protein. Here the first evidence of a direct interaction of F12/E2 with the kinesin-1 complex through an interaction between E2 and the C-terminal tail of KLC2 is presented. Whether this interaction is direct or via other cellular or viral proteins remains to be determined.

In summary, data presented here demonstrate a role for the F12/E2 complex in kinesin-1-mediated IEV trafficking and reveal that this complex binds to KLC isoform 2 via the C-terminal region that varies between different KLC isoforms. The F12/E2 complex represents the second example of a KLC-binding protein with specificity for the KLC2 C-terminal tail and the first example of a virus protein with this specificity.

## Materials and Methods

### Plasmids

pCIneo-Flag-KLC1A (pFlag-KLC1) and pCIneo-Flag-KLC2 (pFlag-KLC2) have been described [52,71]. These plasmids express N-terminal Flag-tagged full length versions of murine kinesin-1 light chain isoform 1A (accession number NM_008450.2) and 2 (accession number NM_008451.2) respectively.

Chimeric KLC1/2 alleles were generated by splicing by overlap extension [72] (using the combination of primers listed in S1 and S2 Tables). Briefly the 5’ and 3’ fragments were generated by PCR using a high fidelity DNA polymerase (platinum *Pfx*, Invitrogen) with short complementary overlapping regions. These were spliced together and amplified using overlapping PCR and cloned into the *Eco*RI and *Xba*I sites of pCI-neo (Promega).

An E2 open reading frame (ORF, codon optimised for expression in human cells, GeneArt), was subcloned into the *Not*I-*Xba*I restriction site of pcDNA3-HA and pcDNA3-V5. These plasmids are two pcDNA3 (Invitrogen) variants containing the coding sequence for either an N-terminal HA-epitope tag with alanine linker (MYPYDVPDYAAAA) or a V5-epitope tag with alanine linker (MGKPIPNPLLGLDSTAAA) inserted into the *Eco*RI-*Not*I site.

### Cells and viruses

The human embryonic kidney cell line HEK 293T (ATCC CRL-11268), human osteosarcoma cell line U-2 OS (ATCC HTB-96) and the African green monkey kidney cell lines BS-C-1 (ATCC CCL-26) and CV-1 (ATCC CCL70) were maintained in DMEM (Gibco Invitrogen) supplemented with 10% heat-treated (56°C, 1 h) foetal bovine serum (FBS). HeLa cells (ATCC CCL-2) were maintained in MEM (Gibco Invitrogen) supplemented with non-essential amino acids and 10% FBS. RK-13 cells were maintained in MEM supplemented with 10% FBS.

All wild type and recombinant viruses were derivatives of VACV strain Western Reserve (WR). The viruses expressing a GFP-tagged capsid protein (A5) have been described (vA5-GFP [20], vA5GFP-ΔA36 and vA5GFP-ΔF12 [41]). Virus stocks were amplified in RK-13 cells (ATCC CCL-37) and titrated by plaque assay on BS-C-1 cells. Viruses used for the flow cytometry egress assay were purified by centrifugation through a 36% (w/v) sucrose cushion as described [33].

### Construction of vE2-HA

The 680 nucleotides upstream of the VACV WR e2l ORF were amplified by PCR using primers E3F-*Hin*dIII (gaccaagcttacgagcgttctaacgcagag) and HAE2R (cgcggccgcagcgtaatctggaacatcgtatgggtacatctttagagaatatactagtc) incorporating an HA-epitope tag encoding region and a *Not*I site. The e2l ORF and 533 nucleotides downstream were amplified by PCR using primers E1R-*Apa*I-*Bam*HI (gaccgggcccggatcctggcgtctaagatattcttccat) and E2FDC1 (gaacgcggccgcgatgatatctgtcacagatattcgta).This added a *Not*I site to the 5’ end of the ORF allowing in frame splicing to the HA-epitope tag-encoding sequence. The spliced product was cloned into the *Hin*dIII-*Apa*I site of pUC13-EcoGPTmCherry, a derivative of pUC13-EcoGPTEGFP [73] in which the ORF encoding the green fluorescent protein was replaced by an ORF encoding the mCherry red fluorescent protein, to create the plasmid pUC12-EcoGPT-mC-HAE2. This plasmid was transfected into CV-1 cells and infected with VACV WR lacking E2 (vΔE2, [48]), and recombinant viruses were isolated by transient dominant selection as described [73,74]. Recombinant vE2-HA was easily distinguishable from vΔE2 parental virus due to the rescue of a wild-type plaque size phenotype. The presence of the E2-HA allele and the absence of the EcoGPT-GFP in the resolved virus was confirmed by PCR, and E2-HA expression was confirmed by immunoblot analysis.

### Generation of F12-HA expressing cell line HEK TRex-F12co-HA

A HEK 293 cell line inducibly-expressing HA epitope-tagged VACV F12 protein was created using the T-REx system (Invitrogen). T-REx-293 cells (expressing the Tet repressor) were transfected with a pcDNA4/TO plasmid into which the ORF for F12-HA, codon optimised for expression in Human cells (GeneArt), was inserted under control of an inducible promoter. Cells were grown and selected using Blasticidin and Zeocin as per the T-REx manufacturer’s instructions to generate a stable polyclonal cell line. Doxycycline-inducible (0.5 μg/ml) expression of F12-HA was confirmed by immunoblot and Immunofluorescence analysis.

### Immunofluorescence (IF) confocal microscopy

HeLa cells seeded onto glass coverslips (thickness no. 1.5) were transfected with pFlag-KLC1 or pFLAG-KLC2 (when appropriate) and 24 h later were infected with vA5-GFP at 5 plaque forming units (PFU)/cell. Cells were fixed 14 h post infection (hpi) for 30 mins in 4% paraformaldehyde followed by 15 mins in 8% paraformaldehyde (in 250 mM HEPES, pH 7.4), permeabilised with 0.1% Triton X-100 and stained using a polyclonal rabbit anti-Flag antibody (Sigma) or rabbit anti-KLC isoform 2 (AbCam ab95881) and anti-rabbit IgG conjugated with AlexaFluor546 secondary antibodies (Life Technologies). Coverslips were mounted in Mowiol (10% w/v Mowiol4–88 (CalBiochem), 25% v/v glycerol, 100 mM Tris-HCl pH 8.5, 0.5 μg/ml DAPI (4',6-diamidino-2-phenylindole, Sigma). Fluorescence images were acquired using a Zeiss LSM780 confocal laser scanning microscopy system mounted on an AxioObserver.Z1 inverted microscope using a 64x Plan Apochromat objective (NA; 1.4) and Zen (Zeiss, 2011 version) acquisition software. Images were processed and analysed using Zen, ImageJ and Photoshop (Adobe) software.

### Immunoprecipitation (IP)

HEK 293T, HeLa or HEK TRex-F12-HAco cells were seeded in 10-cm dishes and transfected 1 day later with the chosen plasmid using TransIT-LTI transfection reagent (Mirius). If required, cells were infected 24 h later at 5 PFU/cell. Cell lysates were generated 8 or 14 h later as follows: cells were washed once with PBS and lysed in Immunoprecipitation (IP) lysis buffer (10 mM Hepes pH 7.4, 0.25% NP-40, 150 mM NaCl) supplemented with cOmplete Mini EDTA-free protease inhibitor cocktail (Roche). Lysates were clarified by centrifugation (15000 × *g*, 15 mins) to remove insoluble material. For anti-HA immunoprecipitations, lysates were incubated with anti-HA mouse monoclonal antibody (clone HA-7)-conjugated agarose beads (Sigma-Aldrich, A-2095). For anti-Flag immunoprecipitations, lysates were incubated with anti-Flag M2 affinity gel (Sigma-Aldrich). For anti-V5 immunoprecipitations, lysates were incubated with 1:300 dilution of an anti-V5 antibody (see below) and protein G-conjugated FastFlow sepharose beads (GE Healthcare). Immunoprecipitations were incubated on a rotator for 4 h or overnight and then washed 4 times with IP lysis buffer. Beads were collected by centrifugation and re-suspended in Laemmli SDS-PAGE loading buffer and precipitated proteins were analysed by SDS-PAGE (10% or 12% acrylamide as required) and immunoblotting.

### Immunoblotting

Proteins were transferred onto Hybond ECL nitrocellulose membranes (GE Healthcare) and incubated with rabbit polyclonal α-HA (Sigma-Aldrich, H6908), rabbit polyclonal α-Flag (Sigma-Aldrich, F7425), mouse monoclonal α-V5 (clone SV5-Pk1, AbD Serotec), mouse monoclonal 63–90 (α-KLC1/2, kind gift from Professor Scott Brady University of Illinois), rabbit polyclonal α-KLC1 (GeneTex, GTX114510), rabbit α-KLC2 (AbCam, ab95881), rabbit polyclonal α-KIF5B (AbCam, ab5629), mouse monoclonal anti-α tubulin (clone DM1A, Millipore) and mouse monoclonal AB1.1 specific for the VACV protein D8 [54]. Blots were imaged and quantified using IRDye-conjugated secondary antibodies (LI-COR) and a LI-COR Odyssey scanner. The secondary antibody used for the α-V5 blots was a Biotin-SP-conjugated AffiniPure goat anti-mouse IgG light chain specific antibody (Jackson ImmunoResearch) and bound Ig was detected with IRDye-conjugated streptavidin (LI-COR). All blots shown are representative of a minimum of are representative of experiments repeated at least 3 times.

### Measurement of VACV egress by flow cytometry after siRNA treatment

Virion egress has been measured either by counting surface virions by live cell staining with anti- B5 antibody and imaging by confocal microscopy [38,41], or by measuring the total integrated fluorescence intensity by epi-fluorescence microscopy [43]. Here, measurement of surface B5 staining of many cells by flow cytometry was used to assess surface virion levels. First, the relative egress of a panel of viruses, vA5GFP, vA5GFP-ΔA36 and vA5GFP-ΔF12, that are known to produce high, low and negligible levels of surface virions, respectively [41], was measured (Fig. 7C i) and this showed a similar trend to that observed by other methods. Then the effects of siRNA knockdown of KLC1, KLC2 or KLC1 and 2 were measured.

Short interfering RNAs (siRNA) targeting KLC isoform 1 (siKLC1) and 2 (siKLC2) each consisting of a pool of two to five 19–25-nucleotide siRNAs (Santa Cruz Biotech) were transfected into HeLa or U-2 OS cells at 50% confluence using the INTERFERin transfection reagent (Polyplus) at an optimised concentration of 10 nM. The efficiency of RNAi-mediated reduction in protein levels was monitored by SDS-PAGE and immunobloting. U-2 OS cells were infected with vA5-GFP or vA5-GFP-ΔA36 at 5 PFU/ml 48–72 h post siRNA treatment. At various time points live cells were stained for VACV protein B5 [38,41]. Briefly, cells were placed on ice and stained with anti-B5 rat monoclonal antibody (19C2, [75]) or anti-F13 rat monoclonal antibody (15B6, used as isotype control, [75]) for 45 mins. The cells were then washed with cold medium and stained on ice with an AlexFluor647-conjugated anti-rat antibody (Invitrogen). Cells were washed with cold PBS, dissociated using the non-enzymatic AccuEasy cell dissociation buffer, fixed overnight in 4% paraformaldehyde to inactivate any remaining virus and analysed by Flowcytometry using a Cyan ADP MLE (Beckman Coulter, Inc.) and Summit 4.3 for Windows software.

### Virus growth curve analysis

For the single step virus growth curve analysis, U-2 OS cells were infected at 5 PFU/cell with vA5-GFP with or without prior siRNA treatment and the culture medium and cell pellets were harvested from infected cells at 1 h pi (to measure input virus levels) and 16 h pi (after measurable levels of EEV have been released but before significant cell lysis has contributed IMVs to the supernatant fraction). The titre of infectious virus was determined by plaque assay on BS-C-1 cells as described [17,76]. All conditions were carried out in triplicate for statistical analysis using GraphPad Prism 5 software for Windows.

### Plaque size measurement

The size of plaques formed on untreated or siRNA-treated U-2 OS cells was measured as follows. U-2 OS cells were infected 48 h post siRNA treatment with vA5-GFP to give well separated plaques (using ∼40 PFU per 35-mm tissue culture dish containing a monolayer of siRNA treated cells) and left for 3 days with a semi-solid overlay (1.5% carboxymethylcellulose) [17,76]. Cells were washed with PBS and then fixed with 4% paraformaldehyde in PBS. Individual green plaques were imaged using an AxioVert.A1 inverted fluorescence microscope connected to a Zeiss MRc colour camera. The plaque surface area was measured using Zeiss AxioVision software and statistical analysis was done using GraphPad Prism 5 software for Windows. The human U-2 OS osteosarcoma cell line was chosen for these experiments because, like the more traditionally used BS-C-1 cells they form even monolayers of cells producing clear plaques upon infection, but being human they can be efficiently treated using prevalidated commercially available siRNA targeting human mRNAs.

## Supporting Information

S1 Fig Protein sequence alignment of murine and human KLC1 and KLC2. ClustalW2 generated protein sequence alignments comparing murine KLC1 (A) and KLC2 (B) to their human counterparts. Residues are coloured according to their level of conservation (fully conserved residues; black with white text, Physico-chemically similar residues; grey with white text, non-conserved residues; white with black text). Amino acid numbers are given to the right of the alignment. The KHC-interacting coiled-coil region is indicated with a blue bar bellow the alignment, each TPR (numbered I-VI) is indicated with a green bar and the C-terminal tail is marked with a red bar. (C) Table showing alignment statistics (% identity and similarity) and accession number information for the sequences used.
(TIF)

S2 Fig Sequence alignment of KLC1 vs KLC2. ClustalW2 generated protein sequence alignment comparison of murine KLC1 and KLC2 using the murine sequences detailed in S1 Fig. panel C. Residues are coloured according to their level of conservation (fully conserved residues; black with white text, physico-chemically similar residues; grey with white text, non-conserved residues; white with black text). This alignment was used to calculate the conservation score plot shown in Fig. 8A i as detailed in Materials and Methods.
(TIF)

S1 Table primers used for generation of chimeric KLC1/2 alleles. A list of primer sequences used in the construction of chimeric KLC alleles by PCR and splicing by overlap extension.
(DOCX)

S2 Table Sequence alignment of KLC1 vs KLC2. Table showing the components used to construct each of the KLC chimeras listed in column 1, including which template and primer pairs were used for the PCR amplification of each fragment spliced together to produce the full length recombinant KLC encoding allele.
(DOCX)
