# Supplementary figures and images for: Vaccinia Virus Protein Complex F12/E2 Interacts with Kinesin Light Chain Isoform 2 to Engage the Kinesin-1 Motor Complex
Source: PLoS Pathog. 2015 Mar 11;11(3):e1004723. doi: 10.1371/journal.ppat.1004723 (PMC4356562; doi:10.1371/journal.ppat.1004723)

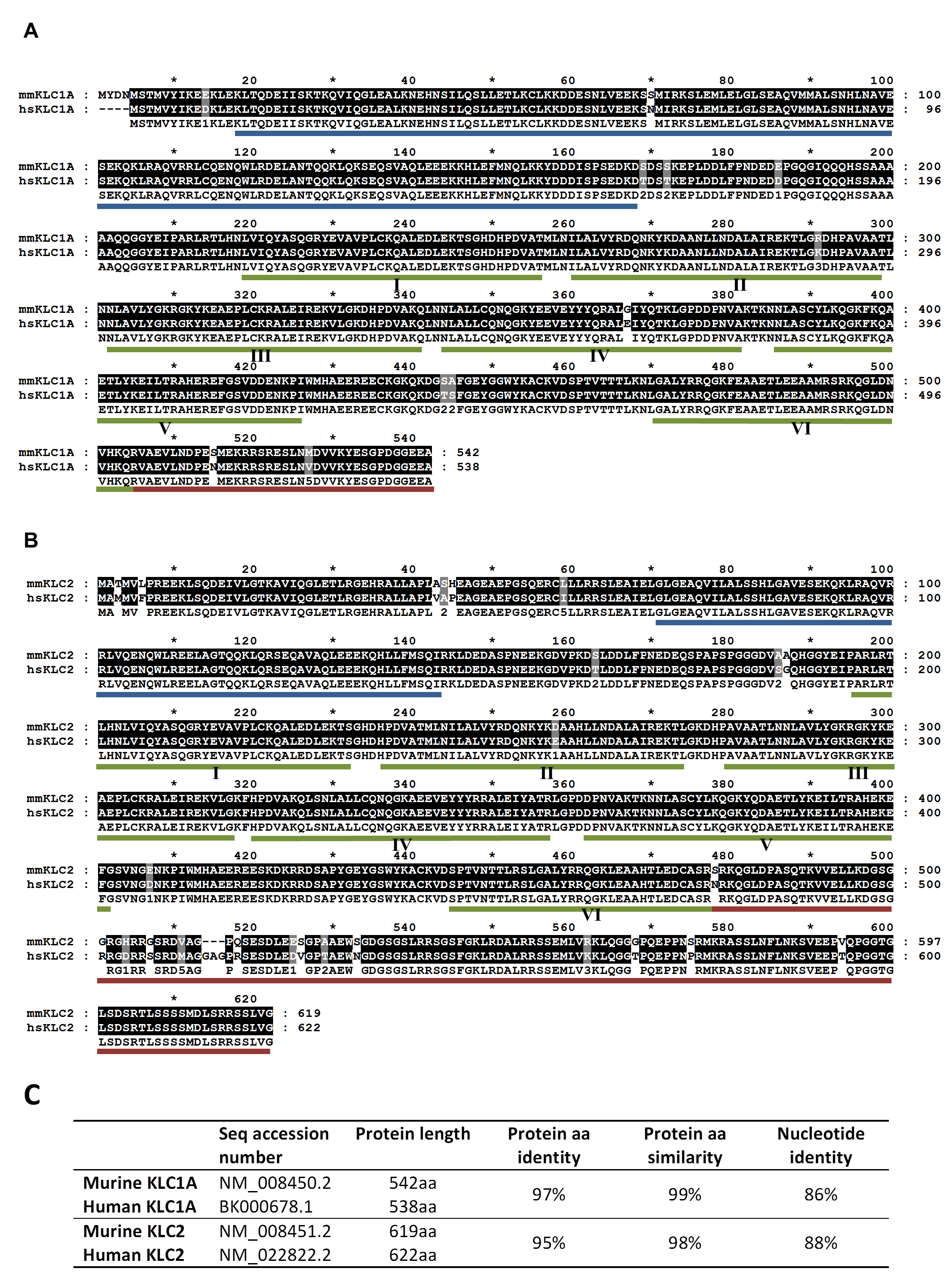

Supplement: S1 Fig — ClustalW2 generated protein sequence alignments comparing murine KLC1 (A) and KLC2 (B) to their human counterparts. Residues are coloured according to their level of conservation (fully conserved residues; black with white text, Physico-chemically similar residues; grey with white text, non-conserved residues; white with black text). Amino acid numbers are given to the right of the alignment. The KHC-interacting coiled-coil region is indicated with a blue bar bellow the alignment, each TPR (numbered I-VI) is indicated with a green bar and the C-terminal tail is marked with a red bar. (C) Table showing alignment statistics (% identity and similarity) and accession number information for the sequences used. (TIF) [file ppat.1004723.s001.tif]

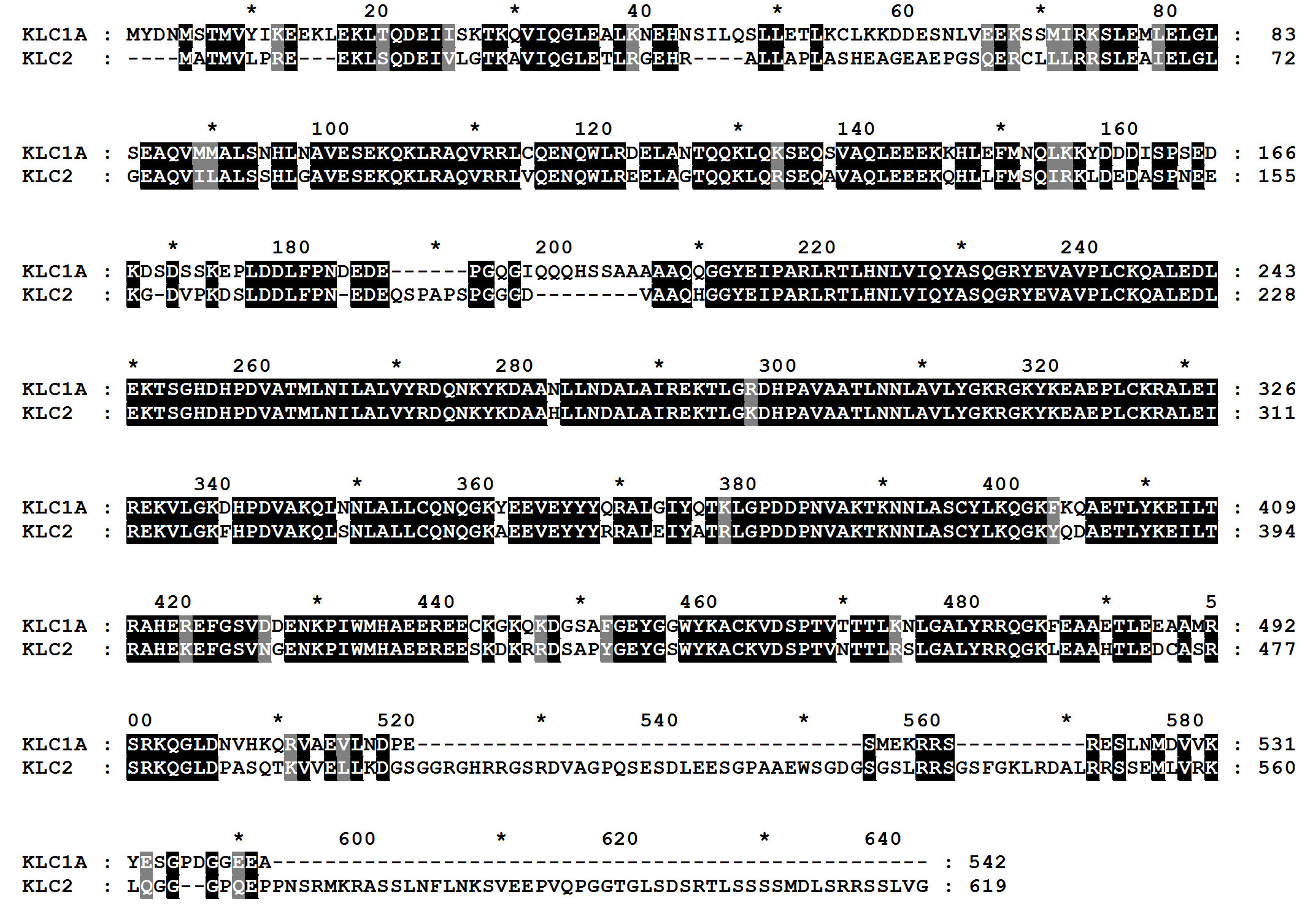

Supplement: S2 Fig — ClustalW2 generated protein sequence alignment comparison of murine KLC1 and KLC2 using the murine sequences detailed in S1 Fig. panel C. Residues are coloured according to their level of conservation (fully conserved residues; black with white text, physico-chemically similar residues; grey with white text, non-conserved residues; white with black text). This alignment was used to calculate the conservation score plot shown in Fig. 8A i as detailed in Materials and Methods. (TIF) [file ppat.1004723.s002.tif]
