# Supplementary material for: Vaccinia Virus Protein Complex F12/E2 Interacts with Kinesin Light Chain Isoform 2 to Engage the Kinesin-1 Motor Complex
Source: PLoS Pathog. 2015 Mar 11;11(3):e1004723. doi: 10.1371/journal.ppat.1004723 (PMC4356562; doi:10.1371/journal.ppat.1004723)
Supplement: S1 Table — A list of primer sequences used in the construction of chimeric KLC alleles by PCR and splicing by overlap extension. (DOCX) [file ppat.1004723.s003.docx]

**S3 supplemental table: primers used for generation of chimeric KLC1/2 alleles**

| **Primer Name** | **Sequence** |
| --- | --- |
| **WG011** | 5' GAT CGA ATT CAT GGA CTA CAA AGA CGA TGA CGA C |
| **WG014** | 5' GAT CTC TAG ATT AGC CCA CCA GGG AGC TT |
| **WG015** | 5' GAT CCA TCT GGG TGA TCC TTT CCC AGA ACC TTC TCC |
| **WG016** | 5' GAT CTG GGA AAG GAT CAC CCA GAT GTG GCC AAG C |
| **WG017** | 5' GAT CCA TCA GGA TGA AAC TTG CCC AGG ACC TTC TC |
| **WG018** | 5' GAT CTG GGC AAG TTT CAT CCT GAT GTT GCC AAA CAG T |
| **WG019** | 5' GAT CTC TAG ACT AGG CTT CCT CCC CTC CG |
| **WG020** | 5' GAT CCA TTG ACT GAT CCA AAC TCC CGC TCG TG |
| **WG021** | 5' GAT CCG GGA GTT TGG ATC AGT CAA TGG AGA GAA CAA GC |
| **WG022** | 5' GAT CCG TCC ACA GAG CCA AAC TCC TTC TCG TGC |
| **WG023** | 5' GAT CGA GTT TGG CTC TGT GGA CGA CGA GAA CAA |
| **WG024** | 5' GAT CCT GTG GGA CTA CTG TCC ACT TTG CAG GCT T |
| **WG025** | 5' GAT CGC AAA GTG GAC AGT CCC ACA GTC AAC ACC ACT |
| **WG026** | 5' GAT CCG GTG GGA CTG TCA ACT TTA CAG GCT TTG TAC CA |
| **WG027** | 5' GAT CTG TAA AGT TGA CCC CAC CGT CAC AAC CAC CTT |
| **WG038** | 5' CCT GTT TGC GCT TAC GTG ACC TCA TGG CG |
| **WG039** | 5' GTC ACG TAA GCG CAA ACA GGG CCT GGA T |
| **WG042** | 5' CAA GAC CCT GGC TGC GGC TGG CAC A |
| **WG043** | 5' CCG CAG CCA GGG TCT TGA CAA TGT TCA CAA |
