# Supplementary material for: Vaccinia Virus Protein Complex F12/E2 Interacts with Kinesin Light Chain Isoform 2 to Engage the Kinesin-1 Motor Complex
Source: PLoS Pathog. 2015 Mar 11;11(3):e1004723. doi: 10.1371/journal.ppat.1004723 (PMC4356562; doi:10.1371/journal.ppat.1004723)
Supplement: S2 Table — Table showing the components used to construct each of the KLC chimeras listed in column 1, including which template and primer pairs were used for the PCR amplification of each fragment spliced together to produce the full length recombinant KLC encoding allele. (DOCX) [file ppat.1004723.s004.docx]

**S4 supplemental table: Details of primer pairs used to generate KLC1/2 chimeras by overlap extension**

|  | **5' fragment** | | | **3' fragment** | | |
| --- | --- | --- | --- | --- | --- | --- |
| **Chimera Name** | **Template** | **Primer 1** | **Primer 2** | **Template2** | **Primer 13** | **Primer 24** |
| KLC1/2A | KLC1A | WG011 | WG014 | KLC2 | WG016 | WG014 |
| KLC1/2B | KLC1A | WG011 | WG020 | KLC2 | WG021 | WG014 |
| KLC1/2C | KLC1A | WG011 | WG024 | KLC2 | WG025 | WG014 |
| KLC2/1A | KLC2 | WG011 | WG017 | KLC1A | WG018 | WG019 |
| KLC2/1B | KLC2 | WG011 | WG022 | KLC1A | WG023 | WG019 |
| KLC2/1C | KLC2 | WG011 | WG026 | KLC1A | WG027 | WG019 |
| KLC1σC-term | KLC1A | WG011 | WG038 | KLC2 | WG039 | WG014 |
| KLC2σTPR6 | KLC2/1C | WG011 | WG038 | KLC2 | WG039 | WG014 |
| KLC2σC-term | KLC2 | WG011 | WG042 | KLC1A | WG043 | WG019 |
| KLC1σTPR6 | KLC1/2C | WG011 | WG042 | KLC1A | WG043 | WG019 |
